# NPC1161B, an 8-Aminoquinoline Analog, Is Metabolized in the Mosquito and Inhibits *Plasmodium falciparum* Oocyst Maturation

**DOI:** 10.3389/fphar.2019.01265

**Published:** 2019-10-25

**Authors:** Timothy Hamerly, Rebecca E. Tweedell, Bernadette Hritzo, Vincent O. Nyasembe, Babu L. Tekwani, N. P. Dhammika Nanayakkara, Larry A. Walker, Rhoel R. Dinglasan

**Affiliations:** ^1^W. Harry Feinstone Department of Molecular Microbiology and Immunology, Johns Hopkins Bloomberg School of Public Health, Baltimore, MD, United States; ^2^Department of Infectious Diseases & Immunology, Emerging Pathogens Institute, University of Florida, Gainesville, FL, United States; ^3^Division of Drug Discovery, Department of Infectious Diseases, Southern Research, Birmingham, AL, United States; ^4^National Center for Natural Products Research, University of Mississippi School of Pharmacy, Oxford, MS, United States

**Keywords:** *Plasmodium*, NPC1161B, malaria, 8-aminoquinoline, transmission-blocking, metabolite analysis

## Abstract

Malaria is a major global health threat, with nearly half the world’s population at risk of infection. Given the recently described delayed clearance of parasites by artemisinin-combined therapies, new antimalarials are needed to facilitate the global effort toward elimination and eradication. NPC1161 is an 8-aminoquinoline that is derived from primaquine with an improved therapeutic profile compared to the parent compound. The (*R*)-(−) enantiomer (NPC1161B) has a lower effective dose that results in decreased toxic side effects such as hemolysis compared to the (*S*)-(+)-enantiomer, making it a promising compound for consideration for clinical development. We explored the effect of NPC1161B on *Plasmodium falciparum* oocyst and sporozoite development to evaluate its potential transmission-blocking activity viz. its ability to cure mosquitoes of an ongoing infection. When mosquitoes were fed NPC1161B 4 days after *P. falciparum* infection, we observed that total oocyst numbers were not affected by NPC1161B treatment. However, the sporozoite production capacity of the oocysts was impaired, and salivary gland sporozoite infections were completely blocked, rendering the mosquitoes non-infectious. Importantly, NPC1161B did not require prior liver metabolism for its efficacy as is required in mammalian systems, suggesting that an alternative metabolite is produced in the mosquito that is active against the parasite. We performed liquid chromatography–mass spectrometry (LC-MS)/MS analysis of methanol extracts from the midguts of mosquitoes fed on an NPC1161B (434.15 *m/z*)-treated blood meal and identified a compound with a mass of 520.2 *m/z*, likely a conjugate of NPC1161B or an oxidized metabolite. These findings establish NPC1161B, and potentially its metabolites, as transmission-blocking candidates for the treatment of *P. falciparum*.

## Introduction

Malaria is a devastating disease that globally affects over 200 million people annually and caused over 435,000 deaths in 2017, mostly in children under the age of five. Despite the significant progress made in recent years, global malaria control has stagnated between 2015 and 2017 (WHO, 2017). *Plasmodium falciparum, Plasmodium vivax, Plasmodium ovale, Plasmodium malariae*, and *Plasmodium knowlesi* are the causative agents of malaria in humans, with *P. falciparum* being the most lethal. *Plasmodia* are transmitted by anopheline mosquito vectors. When the mosquito takes a blood meal, *Plasmodium* sexual stage gametocytes transition into male and female gametes, which fertilize and develop into the motile ookinete. Ookinetes invade the mosquito midgut to form an oocyst, which functions as the sporozoite production site. The development of each oocyst leads to the production of thousands of sporozoites, which then invade the salivary glands of the mosquito, making the mosquito capable of transmitting parasites during its next blood meal (Al-Olayan et al., 2002; Zollner et al., 2006; Nacer et al., 2008).

Current front-line malaria treatments focus on eliminating the asexual blood stages of the parasite; however, even after asexual parasites are eliminated, the gametocytes can persist, allowing transmission to new mosquito vectors (Tangpukdee et al., 2008). A natural bottleneck in parasite number occurs in the mosquito, with a minimum in parasite number occurring in the oocyst stage, making the sexual stage an appealing target for drug intervention to prevent transmission (Vaughan et al., 1992; Porter-Kelley et al., 2006). As the current antimalarials become less effective in Africa and Southeast Asia, alternative antimalarial drugs are needed (German and Aweeka, 2008; Abdul-Ghani et al., 2017). The 8-aminoquinolines (8AQ) have shown potential to fill this need and act as transmission-blocking drugs for malaria (Tekwani and Walker, 2006; WHO, 2011; White, 2013).

The 8AQ are the only class of drug that has been approved by the Food and Drug Administration (FDA) to clear dormant hepatic hypnozoites in patients with *P. vivax* and *P. ovale*. They are also effective against *Plasmodium* asexual and sexual blood stages and mosquito stages (Tekwani and Walker, 2006). This versatility in targeting allows these drugs to act as prophylactics or as treatment/transmission-blocking agents during *Plasmodium* infections (Tekwani and Walker, 2006). For example, primaquine is used as an effective prophylaxis for all forms of malaria, as a treatment for *P. vivax* and *P. ovale* infections, and as a transmission interrupter for *P. falciparum* (Baird and Hoffman, 2004; Hill et al., 2006; Tekwani and Walker, 2006; WHO, 2011; White, 2013). Tafenoquine (WR 238605), a primaquine derivative that was approved by the FDA in 2018 for the radical cure of relapsing malaria, has also been shown to prevent the transition of *P. vivax*, *P. falciparum*, *Plasmodium yoelii*, and *Plasmodium berghei* gametocytes to gametes and inhibit sporozoite production of *P. vivax* and *P. falciparum* (Peters et al., 1993; Ponsa et al., 2003; Walsh et al., 2004). However, these 8AQ induce pronounced hemolytic effects in glucose-6-phosphate dehydrogenase (G6PD)–deficient individuals and may elicit exaggerated responses in NADH methemoglobin reductase-deficient individuals and pose dangers for pregnant women (Hill et al., 2006). Due to these dangers, these drugs cannot provide antimalarial protection for everyone, leaving the need for a safe and efficacious antimalarial (Nanayakkara et al., 2008).

Primaquine and tafenoquine are used as racemic mixtures for the treatment of malaria. Another 8AQ, 8-[(4-amino-1-methylbutyl)amino]-6-methoxy-4-methyl-5-[3,4-dichlorophenoxy]quinoline succinate (NPC1161, WR 233078) in racemic form (NPC1161C) has shown excellent antimalarial activity in animal models (Nanayakkara et al., 2008). In a *P. berghei* model, NPC1161C cleared blood stage parasites and was curative within 3 days of oral treatment at 1 mg/kg/day; by contrast, tafenoquine required 16 mg/kg/day for 3 days, and primaquine was ineffective even at 64 mg/kg/day (Nanayakkara et al., 2008). In the mouse causal prophylaxis model, NPC1161B is active at 1 mg/kg/day for 3 days, while tafenoquine and primaquine require 3 and 20 mg/kg/day for 3 days, respectively (Pybus et al., 2013; Marcsisin et al., 2014). NPC1161C has also shown potent radical curative activity against *Plasmodium cynomolgi* infection in rhesus monkeys (primaquine index 4.8) (Davidson et al., 1981). In some cases, a single enantiomer can provide a better therapeutic index than the racemic mixture (Nguyen et al., 2006). The (*R*)-(−)-enantiomer (NPC1161B) has less hemolytic toxicity in mice and dogs, and more activity against several different parasites in animal models than the racemate (NPC1161C) or the (*S*)-(+)-enantiomer (NPC1161A) (Tekwani and Walker, 2006; Nanayakkara et al., 2008).

Studies have documented the importance of human cytochrome P450 (CYP) enzymes in the metabolism of 8AQ (Ganesan et al., 2009; Myint et al., 2011; Marcsisin et al., 2014). Specifically, enzyme CYP 2D6 has been suggested to play a critical role in the metabolism of primaquine, as well as tafenoquine and NPC1161B, into potential active metabolites (Bennett et al., 2013; Pybus et al., 2013; Marcsisin et al., 2014). Furthermore, NPC1161B had no antimalarial activity in CYP 2D6-knockout mice infected with *P. berghei* (Marcsisin et al., 2014). However, little is known about the metabolism of 8AQ or its potential metabolites in non-vertebrate systems. Although metabolism of other compounds, such as pyrethroids, has been observed in the mosquito vector, no studies have shown the metabolism of 8AQ by mosquitoes (Ibrahim et al., 2016). NPC1161B can prevent exflagellation events in *in vitro P. falciparum* gametocyte cultures, and oocyst development and sporogony in *P. berghei* and *P. falciparum in vivo* systems, suggesting that NPC1161B does not need CYP-mediated metabolism to derive antimalarial transmission-blocking activity (Delves et al., 2012).

Here, we report the use of NPC1161B to disrupt *P. falciparum* sporogonic development in *Anopheles stephensi* mosquitoes with an ongoing *P. falciparum* infection. Additionally, we provide evidence that NPC1161B metabolites are produced in the mosquito midgut following ingestion of NPC1161B-treated blood meal over the 48-h period of blood digestion. 

## Results

### NPC1161B Has A Significant Effect On Oocyst Development

We first determined whether NPC1161B had an effect on oocyst development, and whether metabolism by a vertebrate host was essential for such effects. To address these questions, *An. stephensi* mosquitoes were infected with *P. falciparum* using a standard membrane feeding assay (SMFA). A dose of 10 μM NPC1161B was either provided in the infectious blood meal or 4 days later in a second uninfected blood meal. Previous work demonstrated that the 10 μM dose could inhibit *P. falciparum* gametocyte exflagellation and subsequent oocyst development when added to *in vitro* parasite cultures (Delves et al., 2012). 

**Figure 1:**
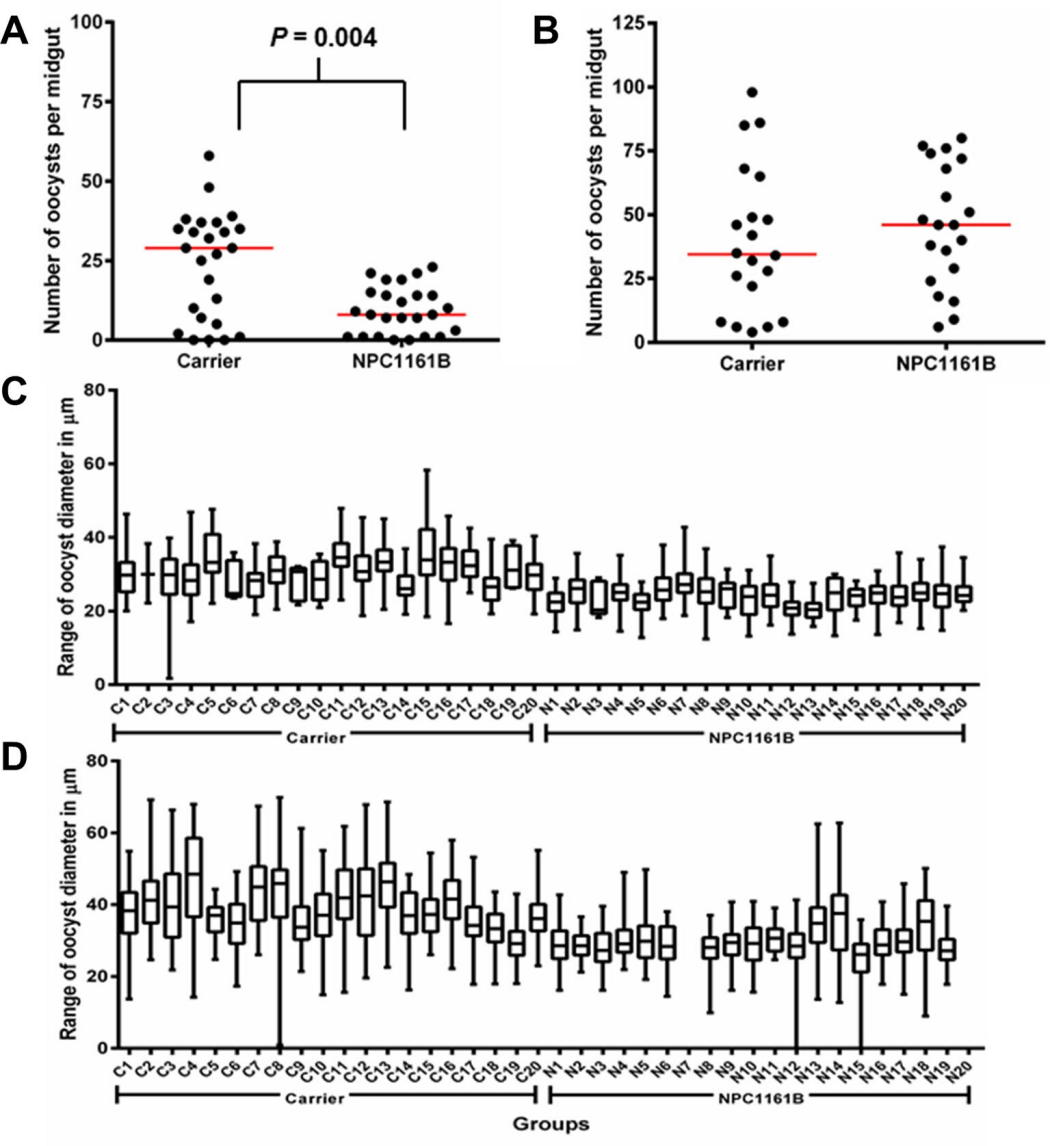
NPC1161B (10 μM) can significantly reduce oocyst number and reduces oocyst size. **(A)** NPC1161B (10 μM) or carrier solvent was administered at the time of infection. The midgut oocyst number was enumerated at day 8 post-infection; a representative replicate is shown. **(B)** NPC1161B (10 μM) or carrier solvent was administered at day 4 post-infection. The oocyst number was enumerated at day 8 post-infection; a representative replicate is shown. **(C**–**D)** NPC1161B (10 μM) or carrier solvent was administered at day 4 post-infection, and oocyst diameter was evaluated at day 8 post-infection. Representative experiments are shown. (N = 20 mosquitoes/group).

On day 8 after infection, mosquito midguts were dissected and either stained with 0.1% mercurochrome for oocyst enumeration or probed with anti-PfCSP antibody and 4′,6-diamidino-2-phenylindole (DAPI) nuclear stain. We observed that NPC1161B, when administered at the time of infection, significantly reduced oocyst intensity (*P* = 0.0040; two-tailed t-test) in the mosquitoes (**Figure 1A**); this is akin to what has been described previously for tafenoquine; however, NPC1161B treatment at day 4 post-infection did not show a significant difference (*P* = 0.4935; two-tailed t-test) between the number of oocysts observed on the control- and NPC1161B-treated midguts (**Figure 1B**), showing that once oocyst development has been initiated (between 24 and 48 h post-infection), NPC1161B does not affect oocyst numbers (Ponsa et al., 2003). We did observe a trend toward a reduction in oocyst diameter with 10 μM NPC1161B treatment on day 4 post-*P. falciparum* infection; however, the statistical significance of this trend was inconsistent (**Figures 1C, D**).

### NPC1161B Affects Sporozoite Development In Oocysts

**Figure 2:**
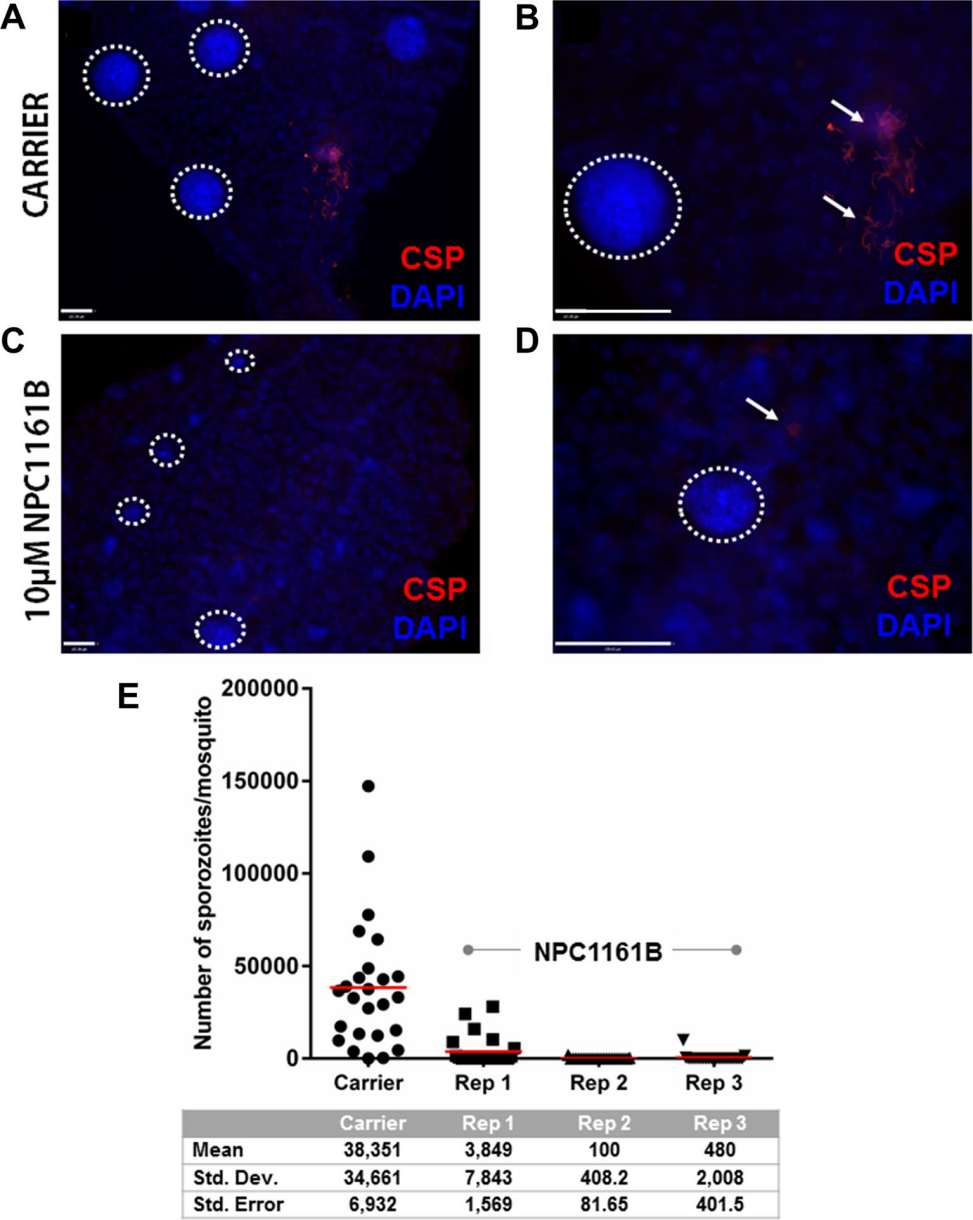
NPC1161B-derived metabolites block sporozoite development. Mosquitoes were given carrier solvent alone **(A-B)** or NPC1161B (10 µM) **(C-D)** at day 4 post-infection. Midguts were dissected on day 11 post-infection. The midguts were probed with anti-*P. falciparum* circumsporozoite protein to identify fully formed sporozoites and stained with DAPI to permit visualization of individual sporozoite segmentation. The dotted white lines demarcate the boundaries of oocysts captured in the field of view. The white arrows point to a group of sporozoites that were released from a burst oocyst and found fixed to the outside of the midgut. **(E)** NPC1161B (10 µM) or carrier solvent was administered to mosquitoes at day 4 post-infection; salivary glands were dissected, and sporozoites were counted at day 14 post-infection. The reduction in sporozoite number for each replicate of NPC1161B treatment (N = 25) as compared to the carrier solvent treatment (N = 25) was found to be statistically significant by ANOVA (*P* < 0.0001).

Having shown that NPC1161B affects oocyst size but did not alter the number when given 4 days post-infection, we set out to study whether NPC1161B would have an effect on the oocyst to sporozoite transition. We administered NPC1161B (10 µM) by membrane feeding at day 4 post-infection and monitored the phenotype of oocyst development using a whole-mount midgut immunofluorescence assay and salivary gland sporozoite enumeration. Oocyst development and maturation on the basal midgut wall are intrinsically asynchronous. As expected, at day 11 post-infection, oocysts that developed without exposure to NPC1161B had already released sporozoites (**Figures 2A, B**). This was not observed in mosquitoes exposed to NPC1161B (**Figures 2C, D**), suggesting that oocyst maturation was arrested. We also observed a wider range of oocyst diameters in the NPC1161B-treated mosquitoes than in the control-treated mosquitoes (**Figures 2A–D**), thus corroborating our previous data (**Figures 1C, D**). DAPI staining of nuclei indicated that, regardless of size, punctate or partitioned DNA was readily observed even in smaller oocysts in the treatment group, indicative of sporoblast initiation (Ganter et al., 2017). However, we did observe weaker PfCSP staining in NPC1161B-treated midguts compared with the carrier-treated controls, suggesting that there was a defect in oocyst maturation or typical developmental pathways. At day 14 post-infection (day 10 post-NPC1161B treatment), when the majority of oocysts should have already ruptured, the salivary glands were dissected and processed, and the number of sporozoites per gland was enumerated. Three blinded replicate treatment cohorts demonstrated a significant reduction (*P* < 0.001 for carrier *vs*. each NPC1161B-treated replicate; two-tailed t-test) in sporozoite number in mosquitoes fed on blood with NPC1161B 4 days post-infection, as compared with control mosquitoes (**Figure 2E**).

### A Novel NPC1161B Metabolite Is Produced In The Mosquito

In light of the finding that NPC1161B is effective at preventing *P. falciparum* development in the mosquito without prior metabolism of the compound by mammalian host CYP enzymes, we hypothesized that there is metabolism of NPC1161B in the mosquito midgut. To understand whether mosquito metabolism of NPC1161B contributes to parasite elimination, we used liquid chromatography–mass spectrometry (LC-MS) to evaluate the metabolites produced in the mosquito midgut after ingestion of a blood meal and exposure to NPC1161B. 

**Figure 3:**
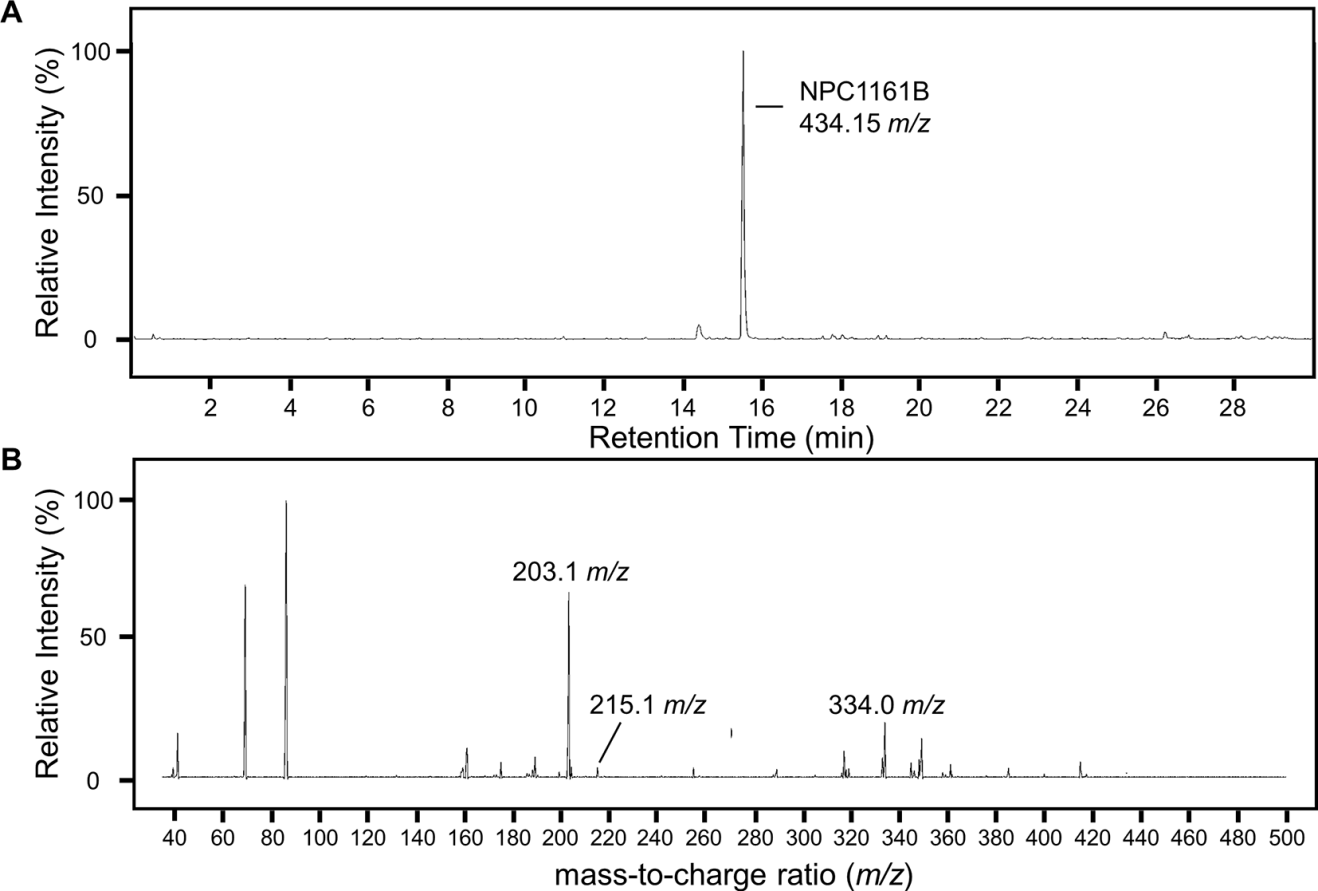
Analysis of NPC1161B standard by LC-QqQ-MS. Extracted ion chromatogram for NPC1161B standard **(A)** and the resulting product ion scan **(B)**. Product ion scanning was used to select fragment ions of NPC1161B for downstream precursor ion scanning to determine metabolites of NPC1161B extracted from mosquito midguts post-feeding. A 1 µl injection of 100 pg/µl was subjected to fragmentation at 42 eV, where Q1 was set to the parent ion (434.15 *m/z*), and Q3 was set to obtain a full scan from 35 to 500 *m/z*. Three ions were selected for use in precursor ion scans: 203.1, 215.1, and 334.0 *m/z*.

To identify potential metabolites of NPC1161B from the midgut, we first analyzed the parent drug by product ion scanning using a triple quadrupole mass spectrometer (QqQ-MS). In this mode, the first quadrupole was used to select for NPC1161B (434.15 *m/z*) eluting from the column, which is fragmented by collision-induced dissociation (CID) in the second quadrupole, and the resulting ions analyzed in full scan mode (35–500 *m/z*) by the third quadrupole. Injections of NPC1161B were repeated in an iterative process to determine the optimal CID energies and resulting fragment ions from the parent drug that could be used to identify metabolites in the blood meal (**Figure 3**).

Midguts (n = 10) from uninfected *An. stephensi* and *An. gambiae* mosquitoes previously fed a blood meal containing NPC1161B (10 µM) were dissected at 48 h post-feeding into cold methanol for metabolite extraction and subsequent analysis by LC-MS. Potential metabolites of NPC1161B produced in mosquitoes were then determined by precursor ion scanning using a QqQ-MS. In this mode, the first quadrupole was set to full scan mode (100–600 *m/z*), the second quadrupole set to fragmentation by CID at 42 eV, and the third quadrupole was set to detect only the fragments generated from NPC1161B with *m/z* of 203.2, 215.1, or 334.0. In this way, only compounds that gave rise to one or more of these fragments were detected. The extracted ion chromatogram (XIC) for the sum of all three product ions shows a number of peaks with low intensity that were not found to stem from metabolized NPC1161B, likely resulting from low abundance of only one or two product ions. However, only a single intense peak was observed and determined to likely be a metabolite of NPC1161B (**Figure S1**). This observation was based on two factors: 1) all three fragment ions were observed and found to stem from a single peak with a mass of 520.2 *m/z*, and 2) the parent ion observed showed a distinctive isotope spacing that results from chlorine-containing compounds, such as NPC1161B, when analyzed by mass spectrometry. 

**Figure 4:**
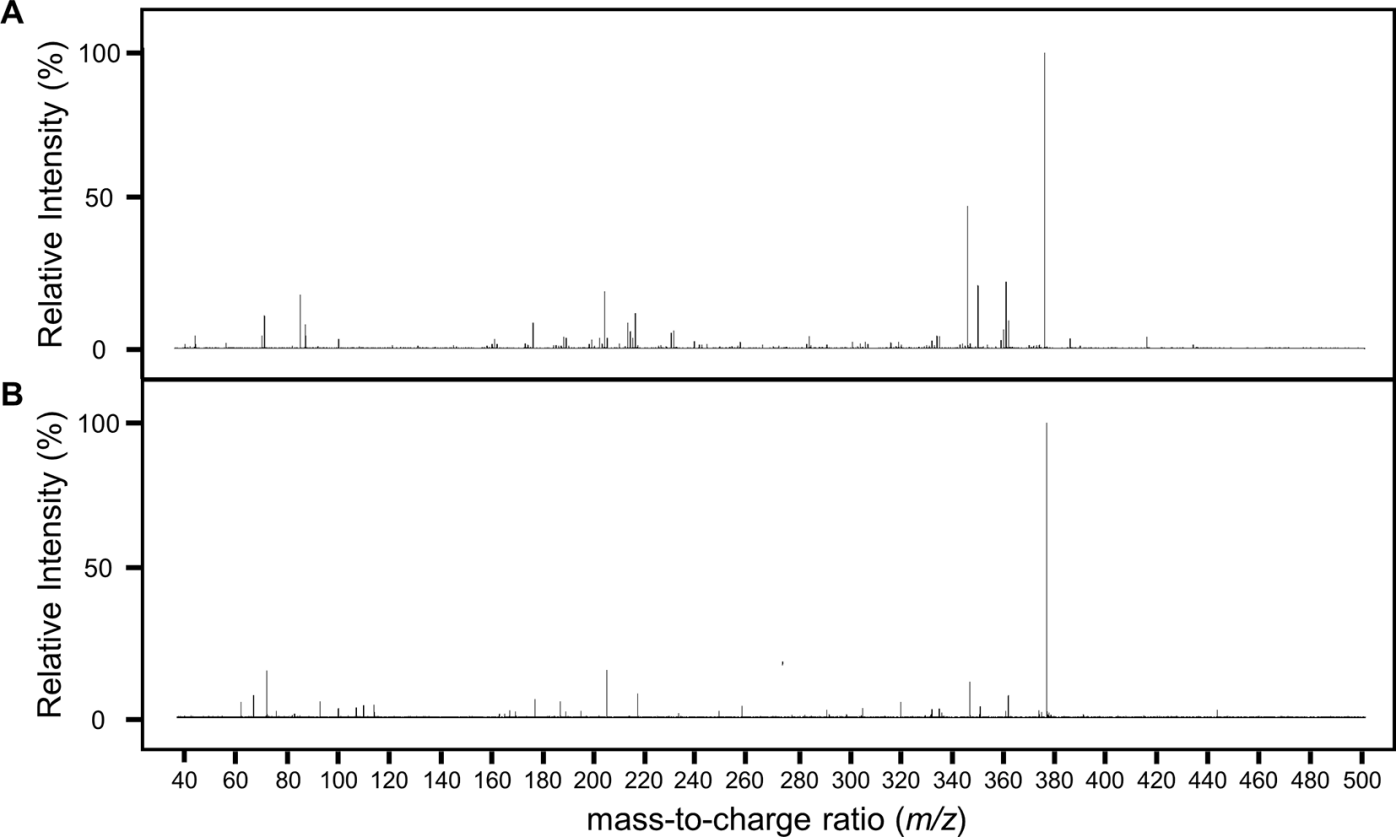
Fragmentation analysis of NPC1161B standard and metabolite by LC-QToF-MS. Mass spectra for NPC1161B standard **(A)** and the metabolite identified from mosquito midgut extracts post-blood meal **(B)** after fragmentation at 42 eV. A number of peaks were found to be shared by both compounds (see **Table 1**).

Having found a potential metabolite of NPC1161B in mosquito midguts, we further characterized this compound using high resolution LC-MS. Both the NPC1161B standard and the previously analyzed midgut metabolite extract were analyzed using quadrupole time-of-flight mass spectrometry (Q-ToF-MS), which produces higher resolution data than the QqQ-MS system used previously. The mass spectra of both the parent compound and resulting metabolite showed the distinctive chlorine isotope spacing expected (**Figure S2**), suggesting that the ion observed in the blood meal was likely a metabolite of NPC1161B produced in the mosquito midgut. Both ions were then subjected to fragmentation by CID, and the resulting patterns showed a high degree of similarity, providing further evidence that the two compounds were related and that the metabolite originated from NPC1161B (**Figure 4**, summarized in **Table 1**). Additionally, to confirm that the observed metabolite from midgut extracts was not the result of direct metabolism by red blood cells (RBCs), as has been seen recently for the related 8-AQ primaquine, we analyzed extracts from uninfected RBCs that were incubated with NPC1161B alone. RBCs were incubated with 10 µM NPC1161B for up to 48 h, then extracts were prepared and analyzed as before (Fasinu et al., 2019). We did not observe the formation of the metabolite previously extracted from midguts (520.2 *m/z*), and in fact, we saw no change in NPC1161B abundance at any of the time points analyzed (1, 6, 24, and 48 h).

**Table 1:** Fragment ion, predicted formula, and the relative intensity for each ion from NPC1161B and its metabolite.

Fragment *m/z*	Predicted formula	% Rel. Int. NPC1161B (434.14 *m/z*)	% Rel. Int. metabolite (520.13 *m/z*)
70.08	[C_5_H_10_]^+^	11.1	16.3
80.95	–	0.9	2.3
84.08	[C_5_H_11_N-H]^+^	17.7	–
86.10	[C_5_H_12_N]^+^	8.0	–
175.09	[C_10_H_10_N_2_O+H]^+^	8.6	6.23
187.06	[C_11_H_9_NO_2_]^+^	3.8	2.2
203.08	[C_11_H_10_N_2_O_2_+H]^+^	19.5	16.7
215.12	[C_13_H_14_N_2_O+H]^+^	11.9	8.35
290.01	–	1.3	1.3
303.04	–	1.6	3.5
318.01	–	2.2	5.3
334.03	[C17H_12_Cl_2_NO_2_+2H]^+^	4.1	1.9
345.06	[C_18_H_14_Cl_2_N_2_O]+H]^+^	51.2	12.0
349.05	[C_17_H_13_Cl_2_N_2_O_2_+2H]^+^	22.0	4.0
360.04	[C_18_H_14_Cl_2_N_2_O_2_]^+^	22.0	13.5
372.05	–	1.0	2.5
375.07	[C_19_H_17_Cl_2_N_2_O_2_]^+^	100	100

**Figure 5:**
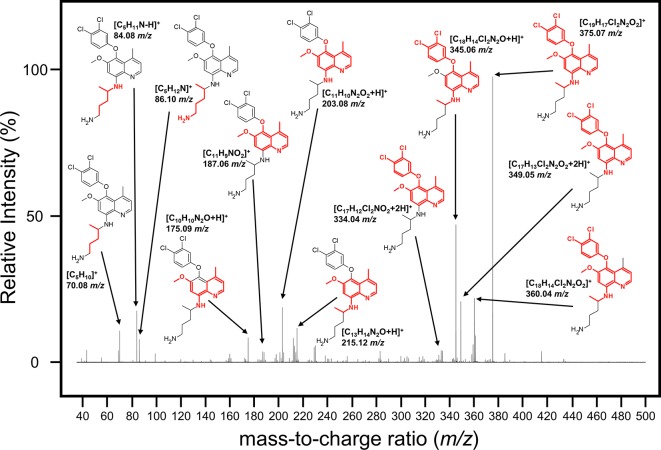
Mass spectrum showing the fragmentation pattern of NPC1161B annotated with the *m/z* and predicted chemical formula with structure. The structure of NPC1161B is shown with the red region denoting the predicted fragment that matches to a given ion.

We next attempted to determine the structure of the NPC1161B metabolite. The structure and resulting fragment ion *m/z* data from NPC1161B were submitted to MetFragWeb, an online tool that performs *in silico* fragmentation and structure prediction (Ruttkies et al., 2016). The algorithm takes as input a structure and fragment ions and produces formula and predicted structures for each fragment ion within a given tolerance. Using the output of this search, we were able to putatively match a number of high abundance fragment ions from NPC1161B with structures and formulas (**Figure 5**; **Table 1**; **Table S1**). Furthermore, many of these fragment ions were also present in the metabolite observed in the midgut extract. While we could not elicit an accurate structure of the metabolite, the major fragment ions of the core 8AQ structure were present. The observed mass of 520.2 *m/z* for the NPC1161B metabolite is significantly higher than the parent compound at 434.15 *m/z*, suggesting this may be a conjugate of NPC1161B, or perhaps a conjugate of an oxidized metabolite. At this stage, structural identification is not possible using conventional methods. Further analysis by NMR spectroscopy is needed to fully elicit a structure for this metabolite.

## Discussion

Transmission-blocking drugs are an essential weapon in the fight to eliminate and eradicate malaria (The malERA Refresh Consultative Panel On Basic Science And Enabling Technologies, 2017). Previous work has established the direct antimalarial effects of NPC1161B through its ability to prevent *P. falciparum* exflagellation and subsequent oocyst development when added directly to gametocytemic blood (Delves et al., 2012). Our study extends these findings to shed new light on the utility of NPC1161B as a transmission-blocking antimalarial that can also be effective when delivered after the establishment of infection in the mosquito. The varied DAPI nuclear staining observed (**Figures 2A–D**), the difference in oocyst size (**Figures 1C, D**), and the significant difference in sporozoite production (**Figure 2E**) in the NPC1161B-treated mosquitoes compared with control-treated mosquitoes suggest that NPC1161B disrupts sporozoite development in the oocyst when given at 10 µm. By preventing the development of sporozoites in the oocyst, no sporozoites can be released into the hemolymph to invade the salivary glands, and the mosquito can no longer transmit the parasite. This suggests that NPC1161B would not only be effective in blocking transmission from patients infected with *Plasmodium* by killing gametocytes but may also be effective when uninfected individuals are fed upon by mosquitoes that are carrying parasites as has been shown previously with the 8AQ tafenoquine (Ponsa et al., 2003).

The mechanism of action of the 8AQ like NPC1161B remains unclear, but it is likely that they exert their antimalarial effects by generating reactive oxygen species and/or interfering with the parasite’s electron transport chain. Historically, these effects were believed to be due to the metabolites from the 8AQ (Grewal, 1981). However, previous work with NPC1161B suggested that it could affect *P. falciparum* without any prior metabolism (Delves et al., 2012). Here, we expanded on this concept and found that NPC1161B can be metabolized uniquely in *An. stephensi* and *An. gambiae* mosquitoes without the need for mammalian CYP enzymes to activate its antimalarial functions. The persistence and dissemination of the active metabolites we observed following ingestion of an NPC1161B-treated blood meal to affect oocysts maturing on the midgut wall are provocative. Interestingly, there was no evidence of the parent compound present in the dissected midguts, suggesting full metabolism, or potentially excretion, within 48 h post-feeding. Determination of the structures of the NPC1161B metabolites identified here (**Figure 4**) could lead to the chemical synthesis of these compounds for subsequent testing in liver stage assays for *P. falciparum* and *P. vivax*, as well as for use in murine malaria *in vivo* models, to determine if potency is maintained while subsequently reducing toxicity (Roth et al., 2018). 

As the 8AQ are generally associated with hemolytic toxicity in G6PD-deficient humans, it would be ideal to find a metabolite that could safely be administered (Hill et al., 2006; Nanayakkara et al., 2008). An additional option to reduce potential toxicity with the parent NPC1161B compound would be to add a modification to prevent activation until it is transferred from the human bloodstream to the mosquito midgut. Recent work has established the feasibility of inserting light-regulated photo switches into drug molecules; in the presence of this photo switch, the compound would only become active when transferred from the darkness of the human bloodstream to the semi-transparent conditions of the mosquito midgut (Nevola et al., 2013; Paaijmans and Fernàndez-Busquets, 2014). Alternatively, infected mosquitoes could be treated directly with NPC1161B, thereby avoiding the potential for toxic side effects in the human host. One potential delivery strategy would be to pretreat mosquito resting surfaces with the compound. Mosquitoes are known to take up atovaquone through their legs, leading to a decrease in or even the absence of oocysts following a subsequent *P. falciparum* blood meal (Tenywa et al., 2017; Maia et al., 2018). However, other compounds such as pyrimethamine are not taken up through the cuticle, and uptake through the legs may hinder proper metabolism and decrease the effectiveness of a compound like NPC1161B. A more practical approach to treat infected mosquitoes is through the use of modified sugar baits containing the drug. Attractive-toxic sugar baits are currently used to deliver insecticides like ivermectin, and they are inexpensive and effective at killing both male and female mosquitoes (Zhu et al., 2017; Sissoko et al., 2019). However, there is concern over the use of insecticides in these baits due to the emergence of resistance in mosquito populations (Tenywa et al., 2017; Maia et al., 2018). An appealing alternative would be to use these baits to deliver drugs that target the pathogen instead of the mosquito. Ingestion of NPC1161B from a sugar meal would likely result in metabolism *via* the midgut, as was seen in our study. This delivery method is more practical than pretreating surfaces but would require further development to ensure drug stability in the field.

In addition to the tolerability profile, it would be ideal for a transmission-blocking drug to have efficacy against not only *P. falciparum* but also against the other human malarial parasites. NPC1161B has already shown efficacy in both *P. falciparum* and *P. berghei*, so it will be important to extend these findings, particularly to *P. vivax* (Delves et al., 2012). Using a drug that can target multiple *Plasmodium* species would be advantageous in the field, especially in areas where cases of malaria are not diagnosed by species-specific rapid diagnostic tests or other species-specific methods.

Overall, there remains a critical need for effective antimalarials on the path toward disease eradication. Currently utilized antimalarials such as chloroquine-, mefloquine-, pyrimethamine-, and artemisinin-based combination therapies are not safe for all patients, neither are they efficacious due to the increasing levels of resistance among parasites (German and Aweeka, 2008; Abdul-Ghani et al., 2017). By disrupting sporozoite development in the oocyst with NPC11161B treatment, sporozoites can no longer invade the salivary glands, therefore ending the cycle of parasite transmission. These findings make NPC1161B a promising compound for further investigation to fill the need for new transmission-blocking antimalarials.

## Methods

### Mosquito Colonies

The *An. stephensi* (SDA500) and *An. gambiae* (KEELE) colonies were raised at 26°C under 70% humidity with equal day–night conditions. Adult mosquitoes were sustained with sucrose and water.

### *P. falciparum* Standard Membrane Feeding Assay

*P. falciparum* (NF54) gametocyte cultures were grown under hypoxic conditions by combining 0.5% asexual mix with 4% hematocrit blood in RPMI culture media with HEPES, hypoxanthine, glutamine, and 10% human serum, which was changed daily during culturing. From day 15 through day 18 post-seeding, the cultures could be used to infect mosquitoes using the standard membrane feeding assay. Approximately 80–100 *An. stephensi* were aspirated into cups and starved overnight to ensure blood feeding. Mosquitoes were at least 4 days old after emerging on the day of infection to optimize feeding but no older than 8 days. All materials used to prepare cultures were warmed to 37°C. Pre-washed blood and culture were centrifuged at 1,800 × *g* for 4 min with the brake on 4 to pellet the uninfected RBCs. The RPMI was aspirated from the packed RBCs, and heat-inactivated human serum (HIHS) was added to 50% hematocrit, all while keeping blood at 37°C. Infected packed RBCs were diluted to 0.3% gametocytemia with 50% hematocrit uninfected blood. Infectious blood remained at 37°C, and 150 μl of infectious blood was added to each sealed feeder. The mosquitoes were allowed to feed for a minimum of 45 min. When most mosquitoes had visibly fed, those that did not feed were removed, and the remainder were provided with sucrose and water along with a saturated cotton ball and filter paper that allowed for oviposition so that the mosquitoes could take another blood meal to receive the drug on day 4 post-infection. Mosquitoes were maintained at 26°C with 70% humidity.

### Administering NPC1161B To Infected Mosquitoes

A 10 mM stock of NPC1161B in carrier consisting of 100 μl of DMSO, 5 μl of TWEEN-80, 400 μl of PEG-400, and 495 μl of sterile de-ionized water was prepared along with the control solution of carrier alone. From the 10 mM NPC1161B stock, 1 μl was added to 1 ml of 50% hematocrit uninfected blood in HIHS for a final concentration of 10 μM NPC1161B. Similarly, 1 μl of carrier control was added to 1 ml of 50% hematocrit uninfected blood in HIHS. On day 4 post-infection with *P. falciparum*, the experimental group of *An. stephensi* mosquitoes was fed on 300 μl of the NPC1161B-treated blood by membrane feeder. Microscopy confirmed that direct addition of 10 μM NPC1161B to the blood did not cause lysis of the RBCs. The control group of *An. stephensi* mosquitoes was fed on 300 μl of the carrier-treated blood by membrane feeder.

To test the blocking capabilities of NPC1161B on *P. falciparum’s* oocyst stage, 1 μl of a 10 mM NPC1161B stock was added to 1 ml of 50% hematocrit infected blood in HIHS with 0.3% gametocytemia for a final concentration of 10 μM of NPC1161B. Similarly, 1 μl of the carrier control was added to 1 ml of 50% hematocrit infected blood in HIHS with 0.3% gametocytemia. The experimental group of *An. stephensi* mosquitoes was fed on 300 μl of the NPC1161B-treated blood by SMFA to be infected and treated simultaneously. The control group of *An. stephensi* mosquitoes was fed on 300 μl of the carrier-treated blood by SMFA to be infected and treated simultaneously.

### Oocyst Quantification

*An. stephensi* midguts were dissected on day 8 post-infection and stained with 0.1% mercurochrome in PBS for 10 min. Then, midguts were washed in PBS, arranged on a 3” × 1” microscope slide in 12 μl of PBS and covered with a 22 × 22 mm cover slip sealed with paraffin. The 5× objective was used with a 10× lens to visualize the midguts and oocysts. Each midgut was imaged with Progress Capture Pro software, ensuring oocysts in all planes were visible. ImageJ and ProgRes CapturePro Software were used to count oocyst number per midgut and to measure oocyst diameter. All data were blinded. Mann–Whitney and unpaired t-tests were run using GraphPad (GraphPad Software, Inc., La Jolla, CA).

### Sporozoite Quantification

The salivary glands of each mosquito were dissected into 100 μl of PBS on day 14–15 post-infection. Each sample was centrifuged at 1,200 × *g* for 3 min and homogenized to release the sporozoites from the salivary glands using a sterile micro-pestle. Samples were vortexed and centrifuged again. Sporozoites were counted blindly on a hemocytometer by averaging two fields. Kruskal–Wallis and two-tailed t-tests were run using GraphPad.

### Immunofluorescence Assay Of Whole Midguts

*An. stephensi* midguts were dissected on day 12 post-infection and fixed in 4% paraformaldehyde for 1 h on ice or overnight at 4°C. Midguts were incubated with 3% bovine serum albumin (BSA) and 0.1% Triton-X-100 to block and permeabilize for 1 h at room temperature or overnight at 4°C. Samples were washed in PBS and incubated overnight at 4°C in a 1:1,000 dilution of 0.5 mg/ml mouse anti-*P. falciparum* circumsporozoite protein [PfCSP; 2A10; MRA183A, Malaria Research and Reference Reagent Resource Center (MR4), Bei Resources], in 3% BSA. Following incubation with the primary antibody, midguts were washed in PBS and incubated for 1 h in a 1:1,000 dilution of 2 mg/ml Alexa Fluor goat anti-mouse 594 antibody (Life Technologies, Frederick, MD) in 3% BSA while being protected from light. Following incubation with the secondary antibody, midguts were washed in PBS, deposited on a slide in a drop of ProLong Diamond Antifade Reagent with DAPI (Life Technologies, Frederick, MD), and covered with a 22 × 22 mm coverslip. Slides were viewed and imaged using a Nikon Upright E800, Nikon 90i, or Zeiss Axioskop2 microscope and the GNU Image Manipulation Program (GIMP).

### Determination Of NCP1161B Metabolites

*An. stephensi* and *An. gambiae* mosquitoes were fed in an SMFA using blood dosed with 10 µM NPC1161B or an equal volume of drug carrier. Forty-eight hours after blood feeding, both drug-treated and control midguts were dissected into tubes containing cold HPLC-grade methanol (MeOH) and placed on ice. Following dissection, metabolites were extracted from midguts (n = 10) by sonication with a microtip probe. Sonication parameters consisted of three pulses of 10 s with a 30 s rest period between each, power set to 20%, and a frequency setting of 7 (Branson Sonifier 450). The samples were kept on ice throughout the sonication process to minimize heat degradation. Following sonication, an equal volume of cold HPLC-grade water was added to each tube, and the sample was vortexed vigorously. Proteins were precipitated following the addition of cold acetone and overnight incubation at −80°C. Samples were then centrifuged at 17,000 × g for 5 min to pellet the proteins; the supernatant was transferred to new tubes, dried using a SpeedVac, and stored at −80°C until analysis. RBCs were also incubated with NPC1161B alone for up to 48 h; then, metabolite extraction was performed using the same protocol to control for potential metabolism of the drug unrelated to the midgut.

Prior to analysis of midgut-extracted metabolites, optimization of ion transitions for the parent compound NPC1161B was performed using product ion mass spectrometry. A standard solution of NPC1161B at 1 mg/ml concentration was serially diluted into 50% MeOH, then injected onto an Agilent RRHT C18 column (50 mm × 2.1 mm) using an Agilent 1290 Infinity II UHPLC system consisting of binary pump, autosampler, and temperature-controlled column compartment. The autosampler maintained the samples at +4°C during analysis, and the column was kept at 30°C. Solvents consisted of water + 0.1% formic acid (FA) as solvent A and acetonitrile (ACN) + 0.1% FA as solvent B, with a flow rate of 600 µl/min. Compounds were eluted from the column using a gradient program as follows: 0–0.5 min 2% B, ramping to 95% B over 3.5 min, and holding at 95% B for 0.5 min before returning to 2% B for re-equilibration, with a total time of 5 min. Eluted compounds were analyzed by an Agilent 6495 triple quadrupole mass spectrometer (QqQ-MS). The system was operated in positive ion mode, with a source temperature of 250°C, gas flow of 15 L/min, sheath gas temperature of 350°C, sheath gas flow of 11 L/min, and capillary and nozzle voltages of 3,500 V and 500 V, respectively. Product ion scan mode was used, with the optimization of parameters for collision energy and selection of fragment ions determined using Agilent Optimizer Software. The first quadrupole was set to select for the precursor ion 434.2 *m/z* with an isolation width of 0.7 *m/z*, while the third quadrupole was set to full scan mode between 35 and 500 *m/z*. The entire system was controlled using Agilent MassHunter Acquisition software.

Analysis of metabolites extracted from midguts was performed using the same system as described above, with the following changes. A Waters Acquity HSS C18 column (150 mm × 2.1 mm) was used, the column was maintained at 50°C, and the following gradient was used: 0–2 min 2% B, ramping to 95% B over 24 min, holding at 95% B for 2 min before returning to 2% B for re-equilibration, with a total time of 30 min. The solvent system and flow were as described above. Source parameters were the same as above, but precursor ion scan mode was used. The first quadrupole was set to full scan mode between 100 and 600 *m/z*, while the third quadrupole was set to selectively monitor three ions representing fragments of the parent compound NPC1161B (203.2, 215.1, and 334.0 *m/z*, respectively). CID with N_2_ gas was done at 42 eV, with a cell accelerator voltage of 5 V, as optimized previously on the standard. A dwell time of 200 ms for each product ion was used, and the fragmenter was fixed at 380 V.

High resolution mass spectrometry analysis for NPC1161B and midgut metabolites was performed using the same LC system, column, and gradient as above. Eluted compounds were analyzed by an Agilent 6550 quadrupole time-of-flight mass spectrometer (Q-ToF-MS). The system was operated in positive ion mode, with the same source parameters as described above. Targeted MS/MS was used to collect fragmentation data with the following parameters: MS1 data were collected from 50 to 1,000 *m/z* at 1 spectra/s, and MS2 data were collected from 35 to 500 *m/z* at 3 spectra/s. Fragmentation was done by CID at 42 eV as before, with the quadrupole isolation width set to 1.3 *m/z*. Both 24- and 48-h post-blood feedings were analyzed, but we found that the parental compound NPC1161B remained in high abundance after 24 h. We therefore focused our analyses at 48 h, which coincides with the physiological completion of midgut blood digestion and is likely when the majority of NPC1161B metabolites can be found in the midgut.

## Data Availability Statement

All datasets that were both generated and analyzed for this study are included in the article/**Supplementary Material**.

## Ethics Statement

The human blood used for mosquito blood meals was collected from a pool of pre-screened donors under an IRB-approved protocol at Johns Hopkins University (Protocol NA00019050) or obtained commercially from anonymous donors through Interstate Blood Bank, making informed consent not applicable.

## Author Contributions

TH, RT, BH, VN, and NN performed the experiments. TH, RT, BH, BT, LW, and RD analysed the data. LW, BT, TH, and RD designed the study. TH, RT, BH, LW, and RD wrote the manuscript. All authors critically reviewed the manuscript before submission.

## Funding

This project was funded in part by the Bloomberg Family Foundation and the University of Florida Emerging Pathogens Institute (RD) and by the US Dept. of Defense, award W81XWH-10-2-0059 to the University of Mississippi (LW). The views expressed herein are those of the authors and do not represent any official position of the Department of Defense.

## Conflict of Interest

The authors declare that the research was conducted in the absence of any commercial or financial relationships that could be construed as a potential conflict of interest.
