# Supplementary material for: NPC1161B, an 8-Aminoquinoline Analog, Is Metabolized in the Mosquito and Inhibits Plasmodium falciparum Oocyst Maturation
Source: Front Pharmacol. 2019 Oct 25;10:1265. doi: 10.3389/fphar.2019.01265 (PMC6823860; doi:10.3389/fphar.2019.01265)
Supplement: Supplementary file 1 [file Table_1.docx]

**Supplemental Table 1.** Fragment ion, predicted formula, and the relative intensity for each ion for NPC1161B and metabolite. For ions with a predicted formula, the predicted fragment structure resulting from NPC1161B is shown in red.

| **Fragment *m/z*** | **Predicted Formula** | **% Rel. Int. NPC1161B (434.14 *m/z*)** | **% Rel. Int. Metabolite (520.13 *m/z*)** | **Predicted Fragment Structure** |
| --- | --- | --- | --- | --- |
| 70.08 | [C_5_H_10_]^+^ | 11.1 | 16.3 |  |
| 80.95 |  | 0.9 | 2.3 |  |
| 84.08 | [C_5_H_11_N-H]^+^ | 17.7 | - |  |
| 86.10 | [C_5_H_12_N]^+^ | 8.0 | - |  |
| 175.09 | [C_10_H_10_N_2_O+H]^+^ | 8.6 | 6.23 |  |
| 187.06 | [C_11_H_9_NO_2_]^+^ | 3.8 | 2.2 |  |
| 203.08 | [C_11_H_10_N_2_O_2_+H]^+^ | 19.5 | 16.7 |  |
| 215.12 | [C_13_H_14_N_2_O+H]^+^ | 11.9 | 8.35 |  |
| 290.01 |  | 1.3 | 1.3 |  |
| 303.04 |  | 1.6 | 3.5 |  |
| 318.01 |  | 2.2 | 5.3 |  |
| 334.03 | [C_17_H_12_Cl_2_NO_2_+2H]^+^ | 4.1 | 1.9 |  |
| 345.06 | [C_18_H_14_Cl_2_N_2_O]+H]^+^ | 51.2 | 12.0 |  |
| 349.05 | [C_17_H_13_Cl_2_N_2_O_2_+2H]^+^ | 22.0 | 4.0 |  |
| 360.04 | [C_18_H_14_Cl_2_N_2_O_2_]^+^ | 22.0 | 13.5 |  |
| 372.05 |  | 1.0 | 2.5 |  |
| 375.07 | [C_19_H_17_Cl_2_N_2_O_2_]^+^ | 100 | 100 |  |
