# Supplementary material for: NPC1161B, an 8-Aminoquinoline Analog, Is Metabolized in the Mosquito and Inhibits Plasmodium falciparum Oocyst Maturation
Source: Front Pharmacol. 2019 Oct 25;10:1265. doi: 10.3389/fphar.2019.01265 (PMC6823860; doi:10.3389/fphar.2019.01265)
Supplement: Supplementary file 2 [file Image_1.pdf]

Supplementary Material

1 Supplementary Data

Supplementary Material should be uploaded separately on submission. Please include any supplementary data, figures and/or tables. All supplementary files are deposited to FigShare for permanent storage and receive a DOI.

Supplementary material is not typeset so please ensure that all information is clearly presented, the appropriate caption is included in the file and not in the manuscript, and that the style conforms to the rest of the article. To avoid discrepancies between the published article and the supplementary material, please do not add the title, author list, affiliations or correspondence in the supplementary files.

2 Supplementary Figures and Tables

For more information on Supplementary Material and for details on the different file types accepted, please see here. Figures, tables, and images will be published under a Creative Commons CC-BY licence and permission must be obtained for use of copyrighted material from other sources (including re-published/adapted/modified/partial figures and images from the internet). It is the responsibility of the authors to acquire the licenses, to follow any citation instructions requested by third-party rights holders, and cover any supplementary charges.

2.1 Supplementary Figures

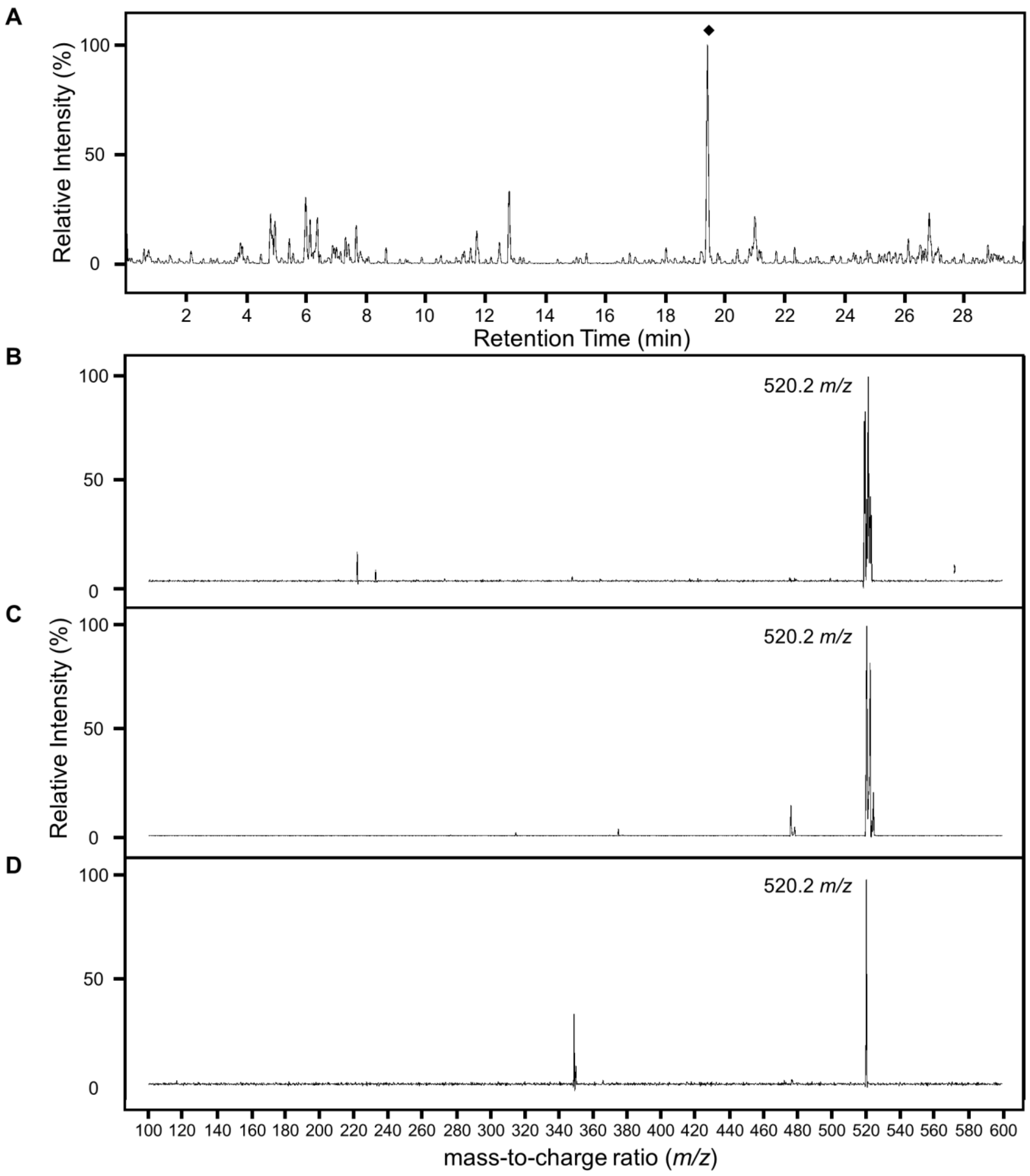

Supplemental Figure S1. Analysis of mosquito midgut extracts after feeding with a blood meal containing NPC1161B by LC-QqQ-MS. (A) Extracted ion chromatogram summed from the three product ions previously determined to result from NPC1161B. (B-D) The resulting precursor ion

scans for the peak denoted by a diamond are shown for each of the three fragment ions (203.1, 215.1, and 334.0 *m/z* respectively). Precursor ion scanning was used to determine potential metabolites of NPC1161B extracted from mosquito midguts post feeding. Extracted metabolites were subjected to fragmentation at 42 eV, where Q1 was set to obtain a full scan from 100-600 *m/z*, and Q3 was set to monitor for the appearance of ions at 203.2, 215.1, and 334.0 *m/z*. The peak denoted by the diamond was the result of fragmentation of a single ion, 520.2 *m/z*.

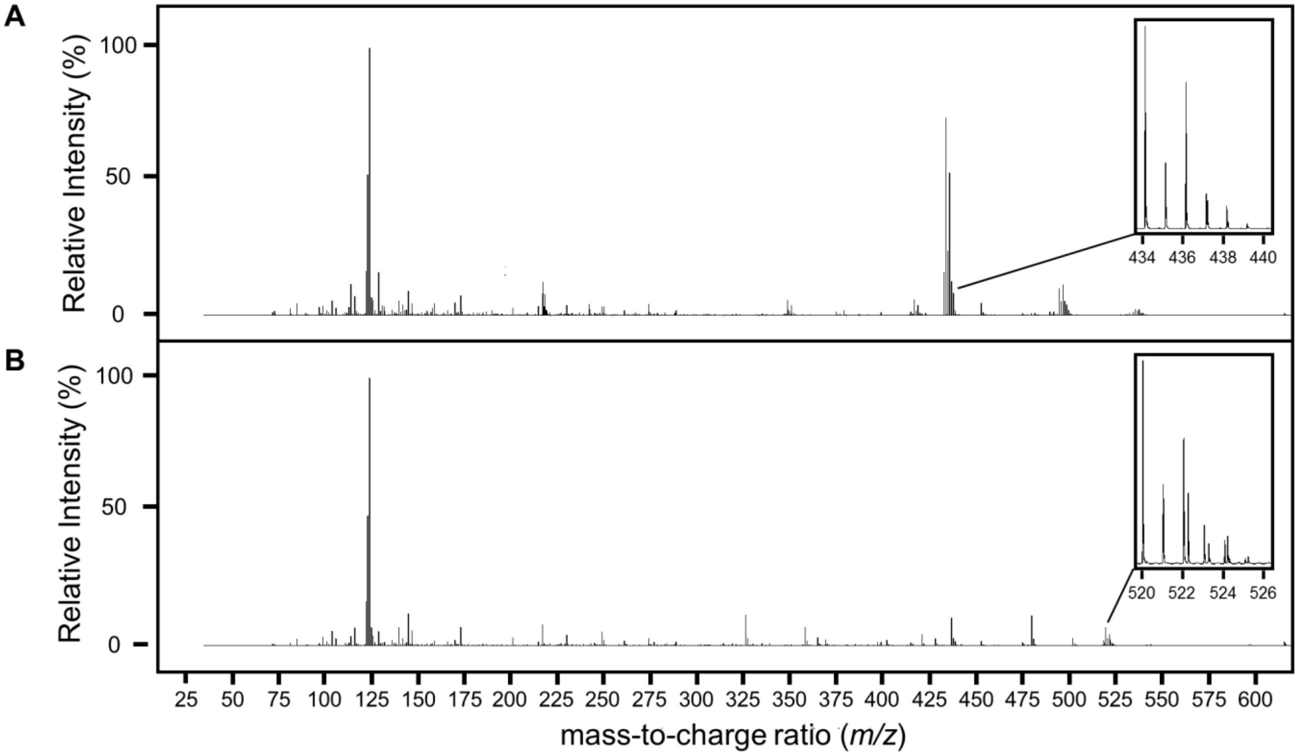

Supplemental Figure S2. Analysis of NPC1161B standard and metabolite by LC-QToF-MS. Extracted ion chromatogram for NPC1161B standard (A) and the metabolite identified from mosquito midgut extracts post blood meal (B). The inset in (A) and (B) are enlarged from the mass range corresponding to each ion and show the distinct chlorine isotope spacing resulting from ^{35}Cl and ^{37}Cl found in NPC1161B, further confirming the ion observed in the midgut extract is a metabolite of the parent drug.
